# Supportive care of patients diagnosed with high grade glioma and their carers in Australia

**DOI:** 10.1007/s11060-022-03991-z

**Published:** 2022-04-09

**Authors:** Georgia K. B. Halkett, Melissa N. Berg, Davina Daudu, Haryana M. Dhillon, Eng-Siew Koh, Tamara Ownsworth, Elizabeth Lobb, Jane Phillips, Danette Langbecker, Meera Agar, Elizabeth Hovey, Rachael Moorin, Anna K. Nowak

**Affiliations:** 1 Faculty of Health Sciences, Curtin School of Nursing, Curtin University, GPO Box U1987, Bentley, WA 6005 Australia; 2 School of Nursing and Midwifery, The University of Notre Dame Australia, Fremantle, WA Australia; 3 Medical School, University of Western Australia, Nedlands, WA Australia; 4 Psycho-Oncology Cooperative Research Group, School of Psychology, Faculty of Science, University of Sydney, Camperdown, NSW Australia; 5 Centre for Medical Psychology & Evidence-Based Decision-Making, School of Psychology, Faculty of Science, University of Sydney, Camperdown, NSW Australia; 6 South West Sydney Clinical School, UNSW Medicine, University of New South Wales, Liverpool, NSW Australia; 7 Liverpool and Macarthur Cancer Therapy Centres, Liverpool, Australia; 8 Ingham Institute for Applied Medical Research, Liverpool, Australia; 9 School of Applied Psychology & The Hopkins Centre, Menzies Health Institute Queensland, Griffith University, Brisbane, QLD Australia; 10 Calvary Health Care Kogarah, Sydney, NSW Australia; 11 School of Medicine, The University of Notre Dame, Sydney, NSW Australia; 12 Faculty of Health, University of Technology, Ultimo, NSW Australia; 13 Faculty of Health, School of Nursing, Queensland University of Technology, Brisbane, Australia; 14 Centre for Online Health and Centre for Health Services Research, The University of Queensland, Brisbane, Australia; 15 Prince of Wales Clinical School, UNSW Medicine, University of New South Wales, Randwick, Australia; 16 Nelune Comprehensive Cancer Centre, Prince of Wales Hospital, Randwick, Australia; 17 Faculty of Health Sciences, Curtin School of Population Health, Curtin University, Bentley, WA Australia; 18 School of Population and Global Health, University of Western Australia, Nedlands, WA Australia; 19 Department of Medical Oncology, Sir Charles Gairdner Hospital, Nedlands, WA Australia

**Keywords:** Supportive care, High grade glioma, Online survey, Multidisciplinary team, Patients, Carers, Brain tumor cancer health professional psychosocial support

## Abstract

**Purpose:**

This study aimed to: determine the supportive care available for Australian patients with High Grade Glioma (HGG) and their carers; identify service gaps; and inform changes needed to implement guidelines and Optimal Care Pathways.

**Methods:**

This cross-sectional online survey recruited multidisciplinary health professionals (HPs) who were members of the Cooperative Trials Group for Neuro-Oncology involved in management of patients diagnosed with HGG in Australian hospitals. Descriptive statistics were calculated. Fisher's exact test was used to explore differences between groups.

**Results:**

42 complete responses were received. A majority of MDT meetings were attended by a: neurosurgeon, radiation oncologist, medical oncologist, radiologist, and care coordinator. Less than 10% reported attendance by a palliative care nurse; physiotherapist; neuropsychologist; or speech therapist. Most could access referral pathways to a cancer care coordinator (76%), neuropsychologist (78%), radiation oncology nurse (77%), or psycho-oncologist (73%), palliative care (93–100%) and mental health professionals (60–85%). However, few routinely referred to an exercise physiologist (10%), rehabilitation physician (22%), dietitian (22%) or speech therapist (28%). Similarly, routine referrals to specialist mental health services were not standard practice. Nearly all HPs (94%) reported HGG patients were advised to present to their GP for pre-existing conditions/comorbidities; however, most HPs took responsibility (≤ 36% referred to GP) for social issues, mental health, symptoms, cancer complications, and treatment side-effects.

**Conclusions:**

While certain services are accessible to HGG patients nationally, improvements are needed. Psychosocial support, specialist allied health, and primary care providers are not yet routinely integrated into the care of HGG patients and their carers despite these services being considered essential in clinical practice guidelines and optimal care pathways.

**Supplementary Information:**

The online version contains supplementary material available at 10.1007/s11060-022-03991-z.

## Introduction

Internationally, more than 330,000 cases of Central Nervous System tumours are diagnosed annually with an age-standardised incidence rate of 4.63 per 100,000 [1]. Five-year relative survival rates are poor, with 22–24% survival for malignant brain and other CNS tumours in Australia and the United States (US) [2, 3]. In this context, we use the term ‘high grade glioma’ (HGG) to encompass Glioblastoma IDH wild type, Astrocytoma IDH mutant (grade 3, 4), and oligodendroglioma IDH-mutant 1p/19q co-deleted (grade 3) [4]. Standard treatments include surgery, radiation therapy, and chemotherapy.

Adults diagnosed with HGG experience functional and neurological deficits, and behavioural and personality changes [5]. Symptom severity ranges from minimal disruption to everyday activities to the patient being fully care-dependent [6]. Consequently, people diagnosed with HGG and their carers experience high levels of distress and have significant unmet supportive care needs [6–8]. Patients and their carers require timely access to support and evidence-based information to manage their disease and its impact [9].

Cancer Care pathways are established to ensure people receive quality cancer care [10]. The Australian Optimal Care Pathway (OCP) for HGG details seven principles of care and a seven-step care pathway encompassing prevention and early detection through to end of life. Supportive care supplements clinical treatment and addresses issues emerging from the cancer diagnosis and treatment. It comprises services, information and resources which meet the individual’s physical, psychological, social, information, and spiritual needs [10]. The OCP pathway highlights the diversity of supportive care needs and importance of access to appropriate supportive care throughout the disease trajectory [10].

The Australian National Brain Cancer Audit concluded that care outcomes will improve if patients and carers have early access to care coordination, rehabilitation and survivorship support services [11]. Multidisciplinary teams and communication between team members, and with patients and carers, facilitate optimal care and ensure patients receive timely and appropriate management [11]. Similarly, international guidelines highlight the importance of providing access to multidisciplinary care, rehabilitation, psychosocial, and allied health support [12]. No previous research has examined the nature of support services for people with HGG and their carers. This study aimed to: determine the supportive care available for patients with HGG and their carers, identify gaps in services and inform best-practice implementation of guidelines and the OCP for this group.

## Methods

### Study design and setting

A cross-sectional online survey documenting supportive care available at clinical sites in Australia was conducted November–December 2018. Ethics approval was granted by Curtin University (HRE2018-0706). The checklist for reporting results of internet e-surveys guided this report [13].

### Recruitment

Multidisciplinary health professionals (HPs) who were members of the Cooperative Trials Group for Neuro-Oncology (COGNO), and involved in management of patients diagnosed with HGG in Australian hospitals were invited to participate.

In 2018 COGNO had approximately 662 members [14]. Surveys were sent to members in clinical disciplines: medical/neuro/radiation-oncology, neurosurgery, nursing, trainees/registrars, allied health (e.g. physiotherapist, occupational therapist, social worker), palliative care, rehabilitation, and psychology. Responses were monitored and reminders targeted to HPs in sites and states with no/low responses. Anecdotal feedback suggested some sites nominated one person to respond or discussed responses as a team as the survey focused on describing what was available to patients at their site as a whole rather than individual experiences in making referrals. Consequently, it was not possible to calculate the response rate.

### Instrument

Questionnaire development was guided by the OCP for people with HGG [15]. The questionnaire was reviewed by representatives from the COGNO Scientific Advisory Committee and Consumer Advisory Panel and piloted by a subset of the sample (n = 11) (Supplement 1 details modifications).

The final questionnaire comprised 38 questions in three sections: (1) socio-demographics; (2) multidisciplinary care; and, (3) multidisciplinary care of carers (Supplement 2). A final question invited open-ended comments about usual care.

#### Socio-demographics

Twelve socio-demographic questions detailed practice location, clinical setting, professional discipline, training, years practicing, new patients treated at site/year, age, and gender.

#### Multidisciplinary care of patients with HGG

This section included 13 questions on: existence and frequency of neuro-oncology multidisciplinary team (MDT) meetings and attending disciplines; hospital or external supportive care services and proportion of patients referred; advice about when to present to general practitioners (GPs); type and frequency of information offered. Supportive care services were grouped by domains aligning with the OCP [15].

#### Multidisciplinary care of carers of patients with HGG

Six questions documented: supportive care services available to carers, proportion of carers referred; advice regarding when to present to GPs; and other types of support available (open-ended response).

### Procedure

Australian COGNO members from eligible disciplines received an email invitation from COGNO to complete the anonymous online questionnaire and forward it to a colleague. The email provided a link to the information sheet, consent form and questionnaire (Qualtrics, Provo, UT). Investigators also disseminated the survey link through their professional networks.

Participants consented online prior to commencing the survey. Two reminders were sent. The pilot survey was distributed on 8 November 2018 and 13 responses received. The survey was modified after feedback (Supplement 1) and redistributed on 29 November 2018 and remained open for 5.6 weeks. Data collected during the pilot was included. For two questions with modified responses, pilot data were manually transformed. Pilot data collected on the proportion of patients referred to services could not be transformed, resulting in some missing data. Two researchers (GH, MB) identified duplicates using demographic responses and for agreed duplicate pairs, earlier responses were removed.

### Data analysis

Analysis was completed using IBM SPSS Version 27. Incomplete surveys were included, with missing data for each section identified. Descriptive statistics were calculated. The OCP states most HGG patients will require specialised supportive care, therefore, we expected at least half of patients would be referred to a particular service [11]. Fisher's Exact Tests (FET) [16] were used to explore differences between groups in cross-tabulations. The degrees of freedom (df) are shown in significant tests where df > 1. A *p* value < 0.05 was deemed statistically significant. Content analysis was used to compile free-text responses.

## Results

Consent was obtained from 55 HPs (n = 13 pilot; n = 42 final); six answered no questions and seven responses were duplicates, leaving 42 responses (n = 5 pilot; n = 37 final). Respondents took < 15 min to complete the survey (IQ_25_ = 7.67 min; IQ_75_ = 14.67 min). Table 1 summarises HP socio-demographic data.

**Table 1:** Personal, professional and workplace characteristics of HP participants

	N = 42 (100%)
Age^a^ (years) Mean (S.D.)	47.4 (9.60)
Range	28.0—72.0
	N (%)
Gender	
Female	25 (60)
Male	15 (36)
Prefer not to answer	2 (5)
Highest level of training	
Bachelor degree	4 (10)
Graduate certificate	2 (5)
Master degree	4 (10)
PhD degree	7 (17)
Medical college fellowship	18 (43)
Medical college fellowship and postgraduate degree	7 (17)
Discipline	
Medical oncologist	15 (36)
Radiation oncologist	10 (24)
Neurosurgeon	7 (17)
Nurse or care coordinator	6 (14)
Allied health (Occupational therapist, social worker)	2 (5)
Palliative care physician	2 (5)
Years practicing in discipline	
≤ 5	4 (10)
6–10	11 (26)
11–15	7 (17)
16–20	10 (24)
≥ 21	10 (24)
Years practicing in current position	
≤ 5	11 (26)
6–10	13 (31)
11–15	11 (26)
≥ 16	7 (17)
Australian state/territory of current workplace	
New South Wales	17 (41)
Victoria	14 (33)
Queensland	7 (17)
South Australia	1 (2)
Western Australia	1 (2)
Australian Capital Territory	1 (2)
Tasmania	1 (2)
Workplace location	
Metropolitan	36 (86)
Regional/rural	6 (14)
Healthcare setting	
Hospital public	31 (74)
Hospital private	2 (5)
Private practice	1 (2)
Both public and private	8 (19)
Type of clinical setting	
Tertiary referral cancer centre	34 (81)
District/local hospital	4 (10)
Non-inpatient cancer treatment centre	4 (10)

^a^ Missing data

### Neuro-oncology multidisciplinary team meetings

Table 2 summarises data about neuro-oncology MDT meetings. Almost all HPs reported their site held neuro-oncology MDT meetings (95%) and most discussed all patients newly-diagnosed with HGG (62%). The following specialist HPs were most frequently reported as attending MDT meetings: neurosurgeon (100%), radiation oncologist (95%), medical oncologist (95%), radiologist (87%), and care coordinator (77%). A neuropathologist (64%) or pathologist (51%) were less likely to attend. Minimal MDT attendance was reported for: palliative care nurse (8%); nuclear medicine physician (8%); physiotherapist (5%); neuropsychologist (5%); and speech therapist (3%).

**Table 2:** Neuro-oncology multidisciplinary team meetings at HP participants' worksite

	N = 42 (100%)	
Neuro-oncology meeting at worksite		
Yes	40 (95)	
No	2 (5)	
Percentage of patients newly-diagnosed with HGG discussed at MDT meetings^a^		
0—50%	7 (19)	
55—95%	7 (19)	
100%	23 (62)	
Frequency of formal MDT meetings^a^		
Weekly	16 (40)	
Fortnightly	16 (40)	
Monthly	7 (18)	
Other	1 (3)	
Disciplines which attend most MDT meetings (either in person or remotely)^a,b^		
Neurosurgeon	39 (100)	
Medical oncologist/neuro-oncologist	39 (100)	
Pathologist/neuropathologist	37 (95)	
Radiation oncologist	37 (95)	
Radiologist	34 (87)	
Care coordinator	30 (77)	
Neurosurgery nurse	15 (38)	
Clinical trials coordinator/staff/researchers	12 (31)	
Neurologist	8 (21)	
Radiation oncology nurse	5 (13)	
Social worker	5 (13)	
Psychiatrist	5 (13)	
Medical oncology nurse	4 (10)	
Occupational therapist	4 (10)	
Palliative care specialist	4 (10)	
Palliative care nurse	3 (8)	
Nuclear medicine physician	3 (8)	
Physiotherapist	2 (5)	
Neuropsychologist	2 (5)	
Speech therapist	1 (3)	
Other^c^		4 (10)

^a^ one HP did not answer this question

^b^ multiple responses allowed

^c^ Other included: radiation therapists (n = 2); MDT coordinator (n = 1); and trainee (n = 1)

A significantly greater proportion of metropolitan HPs had neuro-oncology MDT meetings at their site (100%) compared with HPs working in a regional/rural location (67%; p = 0.017). A significantly greater proportion of HPs working in a tertiary cancer centre had a neuro-oncology MDT meeting (100%) compared with those in a district/local hospital (75%) or non-inpatient cancer treatment centre (75%; df = 2, p = 0.033).

### Multidisciplinary care of patients diagnosed with HGG

#### Physical, psychological and social domains

Supportive care services addressing needs in the physical, psychological and social domains are summarised in Table 3. At their site, a majority of HPs (85%-100%) could refer patients to a physiotherapist, hospital-based palliative care, speech therapist, dietitian, social worker, rehabilitation physician, occupational therapist, domiciliary palliative care service, psychiatrist, or general psychologist. Between 72–78% could refer patients to a cancer care coordinator, neuropsychologist, radiation oncology nurse, or psycho-oncologist. Two-thirds could refer patients starting oral chemotherapy to a pharmacist and 54% could refer to an oral chemotherapy nurse while most HPs (60%-85%) could refer patients to a psychology, psychiatry, or counselling service provider.

**Table 3:** Availability of supportive care services for patients diagnosed with HGG which address needs in the physical, psychological and social domains

At your site can you refer patients diagnosed with HGG to a	Service is available (any location) n (%)	On site provider n (%)	External or private provider n (%)	Service not available at site n (%)	Unsure, do not refer n (%)	Total N = 42^a^(%)
Physiotherapist	41 (100)	38 (93)	3 (7)	0 (0)	0 (0)	41 (100)
Hospital based or inpatient palliative care	41 (100)	38 (93)	3 (7)	0 (0)	0 (0)	41 (100)
Speech therapist	39 (98)	35 (88)	4 (10)	1 (3)	0 (0)	40 (100)
Dietitian	38 (98)	37 (95)	1 (3)	0 (0)	1 (3)	39 (100)
Social worker or welfare officer	38 (95)	36 (90)	2 (5)	1 (3)	1 (3)	40 (100)
Rehabilitation physician	38 (93)	28 (68)	10 (24)	2 (5)	1 (2)	41 (100)
Occupational therapist	37 (93)	32 (80)	5 (13)	1 (3)	2 (5)	40 (100)
Domiciliary palliative care service (may or may not be linked with hospital)	37 (93)	26 (65)	11 (28)	1 (3)	2 (5)	40 (100)
Psychiatrist	35 (85)	29 (71)	6 (15)	4 (10)	2 (5)	41 (100)
General psychologist	34 (85)	25 (63)	9 (23)	3 (8)	3 (8)	40 (100)
Cancer care coordinator/nurse navigator	31 (76)	31 (76)	0 (0)	9 (22)	1 (2)	41 (100)
Neuropsychologist for cognitive function testing (neuropsychological/neuropsychiatric testing)	31 (78)	25 (63)	6 (15)	6 (15)	3 (8)	40 (100)
Radiation oncology nurse	30 (77)	29 (74)	1 (3)	4 (10)	5 (13)	39 (100)
Oncology psychologist	29 (73)	28 (70)	1 (3)	4 (10)	7 (18)	40 (100)
Oral chemotherapy/oncology pharmacist	26 (67)	24 (62)	2 (5)	5 (13)	8 (21)	39 (100)
Counsellor, including telephone service	24 (60)	17 (43)	7 (18)	9 (23)	7 (18)	40 (100)
Exercise physiologist	23 (59)	12 (31)	11 (28)	9 (23)	7 (18)	39 (100)
Neurosurgery nurse	21 (54)	21 (54)	0 (0)	12 (31)	6 (15)	39 (100)
Oral chemotherapy nurse	21 (54)	18 (46)	3 (8)	12 (31)	6 (15)	39 (100)
Nurse practitioner	11 (27)	10 (24)	1 (2)	25 (61)	5 (12)	41 (100)
Other support services	9 (31)	8 (28)	1 (3)	5 (17)	15 (52)	29 (100)
Epilepsy nurse	6 (15)	6 (15)	0 (0)	19 (49)	14 (36)	39 (100)

^a^ each item contains missing data i.e. there were 42 HPs who answered at least one item for this question, but no item was answered by all 42 HPs

More metropolitan HPs could refer patients to a neuropsychologist (metropolitan = 85%; regional/rural = 33%; p = 0.016). Similarly, more HPs who worked in a tertiary cancer centre could refer to a neuropsychologist (84%) or rehabilitation physician (97%) compared with HPs from district/local hospitals (25%; df = 2, p = 0.033; 50%, df = 2, p = 0.042 respectively).

**Fig. 1:**
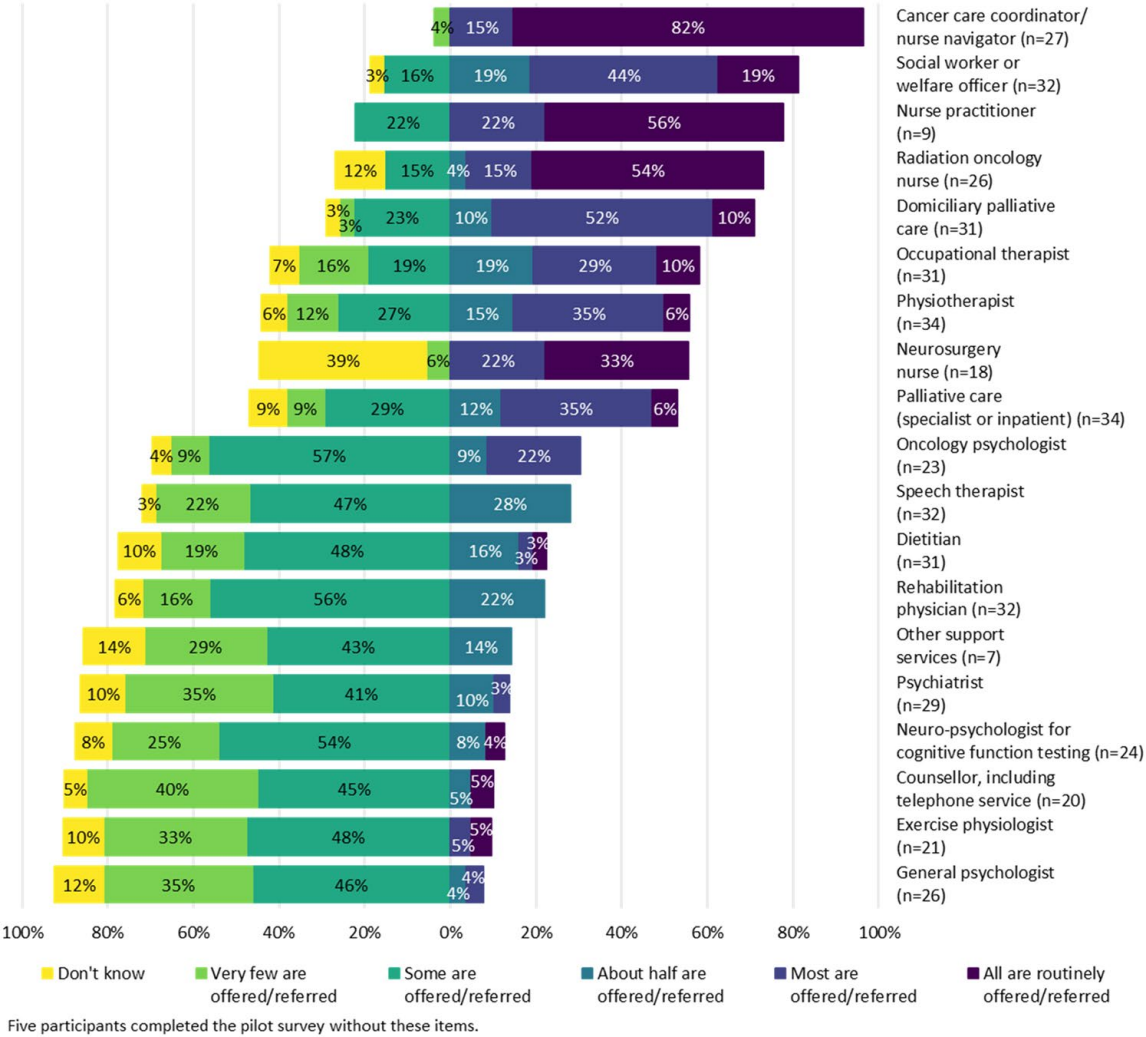
At sites where each service is available, proportion of HGG patients referred to supportive care services which address needs in the physical, psychological and social domains

At sites with each service available, the proportion of HGG patients referred to supportive care services is shown in Fig. 1. Disciplines with infrequent referrals included exercise physiology, rehabilitation physician, speech therapist, or dietitian. Approximately half of HPs stated some/very few patients were referred to an oral chemotherapy/oncology pharmacist. Most HPs stated some/very few patients were referred to mental health services: counsellor (including telephone service), general psychologist, neuropsychologist, psychiatrist, or psycho-oncologist.

Compared to metropolitan HPs, more HPs in a regional/rural location stated at least half of HGG patients were referred to: oral chemotherapy nurse (regional/rural = 100%; metropolitan = 23%; p = 0.007); an occupational therapist (regional/rural = 100%; metropolitan = 48%; p = 0.028); a physiotherapist (regional/rural = 100%; metropolitan = 46%; p = 0.024); or to hospital-based palliative care (regional/rural = 100%; metropolitan = 43%; p = 0.020).

#### Support groups, spiritual, practical and information domains

Spiritual and practical supportive services and support groups were available to most patients (Table 4).

**Table 4:** Availability of spiritual and practical supportive services, complementary service providers, and support groups for patients diagnosed with HGG

At your site, can you refer patients diagnosed with HGG for a…	Service available (any location) n (%)	On site provider n (%)	External or private provider n (%)	Service not available at my site n (%)	Unsure, I do not refer to this service n (%)	Total N = 36 (100%)
Fitness-to-drive assessment	28 (78)	10 (28)	18 (50)	8 (22)	0 (0)	36 (100)
Pastoral care or a person who provides spiritual support	27 (75)	21 (58)	6 (17)	4 (11)	5 (14)	36 (100)
Support with legal issues (advance care planning, power of attorney, wills)	26 (72)	17 (47)	9 (25)	5 (14)	5 (14)	36 (100)
Support group^a^	21 (60)	10 (29)	11 (31)	11 (31)	3 (9)	35 (100)
Complementary therapy service provider^a, b^	11 (31)	7 (20)	4 (11)	15 (43)	9 (26)	35 (100)

^a^ contains missing data

^b^ For example meditation, relaxation, aromatherapy, acupuncture, reflexology, and massage

A significantly greater proportion of HPs from a tertiary cancer centre could refer patients for a fitness-to-drive assessment compared with a district/local hospital (86% vs. 25%; df = 2, p = 0.029).

At sites where spiritual or practical supportive care was available, the proportion of patients referred is shown in Supplement 3 (Fig. 1). Supplement 3 (Table 1) summarises the proportion of patients newly diagnosed with HGG who are given various sources of information.

#### Health issues referred to General Practitioners (GP)

Nearly all HPs (94%) reported HGG patients were advised to present to their GP for pre-existing conditions/comorbidities. Fewer advised patients to see their GP for social issues (36%), mental health (19%), symptoms (11%), cancer complications (11%), and treatment side-effects (3%) (Supplement 4).

### Care of carers

Thirty-five HPs provided data about the multidisciplinary care of carers of patients with HGG. Availability of specialist nursing, psychological, and social supportive services for carers and the proportion of carers referred are shown in Supplement 5 (Table 1 and Fig. 1). Most HPs (62%-83%) could refer carers to a social worker/welfare officer, care coordinator/nurse navigator, or psychologist. Of HPs who answered the question (n = 32), approximately half could refer a HGG patient carer to any support group (Supplement 5–Table 2).

Supplement 5-Table 3 shows the proportion of carers of HGG patients advised to present to their general practitioner at different timepoints during treatment. At least 25% indicated they either did not know or that carers were never advised to see a GP about their 'loved one'. The main reasons that carers might be advised to present to their GP included: psychological support (n = 20), carers health (n = 5), and assistance to complete paperwork for financial support (n = 1).

## Discussion

This study documented the supportive care available and routinely utilised for patients with HGG and their carers. Our survey specifically investigated key recommendations within the OCP, which outlines the nationally agreed best practice for HGG [10].

According to best international evidence, MDT meetings are critical in managing brain tumours [12] and newly-diagnosed patients should have a MDT recommended plan within two weeks of diagnosis or before surgery [10]. Almost all responding sites reported regular neuro-oncology MDT meetings at which most new patients were discussed. Meetings appear most focused on initial treatment planning. Few HPs reported care coordinators, neurosurgery nurses and social workers attended, despite their designation as core members of a neuro-oncology MDT [10]. Professionals from disciplines providing supportive care and psychosocial support rarely attended. Consequently, psychosocial, allied health, rehabilitation, and supportive care needs may be under-addressed [10].

Cancer care coordinators and specialist nurses play a key role in supporting this group [10, 17]. Access to specialist nursing care varied, with a quarter of sites lacking a cancer care coordinator. However, when available, nearly all patients were referred. We identified important potential service gaps including limited access to neurosurgery, oral chemotherapy, and seizure or epilepsy nurses. Despite the prevalence of seizures in people with HGG [17–19], only 15% reported access to a seizure/epilepsy nurse. When available, half of HGG patients were referred, highlighting service relevance. In other settings, care provided by seizure/epilepsy nurses may be associated with reductions in seizure-related Emergency Department (ED) and GP presentations [20, 21].

Best evidence highlights the importance of screening for supportive care needs and providing access to relevant support services [10, 22]. Screening for supportive care needs should begin at presentation and occur regularly [23]. Although we did not capture timing of referral, we documented service availability and the proportion of patients and carers referred to each along the care pathway.

Only some patients were referred to mental health services, with some only available through external providers, presenting an access barrier. Australian clinical practice guidelines and OCPs indicate early consistent psychosocial care of brain cancer patients and their carers is critical [10, 18, 24, 25]. Most cognitive rehabilitation intervention programs reported improvements in patients' cognitive test-performance [26] and could be considered for inclusion in psychosocial care of HGG patients. Future research will provide greater understanding of psychological management of people with brain tumour and their carers, including screening for psychological distress and cognitive deficits, and how these issues are managed in practice. Improved access to psychological interventions through remote delivery is an important consideration. However, telehealth research indicates uptake and adherence are higher for interventions involving real-time interactions rather than self-guided interventions [27].

The OCP emphasises the importance of early allied health referral when required [10]. We found low referral to dietitians, who have a key role in the multidisciplinary care of neuro-oncology patients. Patients with brain tumours often experience weight gain resulting from corticosteroids and mobility limitations, and may benefit from dietitian consultations. We identified low referrals to speech therapists, despite the frequent occurrence of speech aberrations (eg. expressive and receptive dysphasia) [18] and increasing communication deficits over time [28]. Occasionally issues relating to swallowing emerge, which can be addressed by speech pathologists [29].

Rehabilitation plays an integral role in managing symptoms/complications of brain malignancies [30]. Although most HPs indicated availability of a rehabilitation physician, only 20% stated at least half their patients were referred. Patients with brain cancer who receive early intervention make comparable gains and report similar levels of satisfaction with post-surgical rehabilitation to those with stroke [31]. Accordingly, access to cognitive and physical rehabilitation is important to support management of functional loss and activity limitations [23, 32]. Exercise interventions for HGG patients can present challenges due to the burden of symptoms and other health-related commitments. However, they are perceived to be beneficial for patient health, a sense of control and social interaction, and carer respite [33].

Best evidence recommends palliative care is discussed early and, for teams without a palliative care specialist, emphasises the importance of engaging primary care and community palliative care services [10, 34]. Despite the poor prognosis of HGG, involvement of palliative care services was relatively low. Few MDTs were attended by a palliative care clinician. Palliative care services were available at most sites; however, referral was not universal. Only a small proportion of HPs noted all patients were referred to palliative care. A systematic review of palliative care utilisation by glioblastoma patients identified advance care planning for up to half of patients, palliative care referrals and consultations for a third, and hospice referrals for most, with variable hospice use (38–86%) [35]. Although it is unclear from our survey whether low referral rates impacted the use of Advance Care Planning, such planning helps meet patients’ end of life preferences and reduces healthcare costs in patients with cognitive impairment or dementia [36]. The Australian National Palliative Care Standards states palliative care should be available to all patients with an active, progressive, or advanced disease [37] and the OCP for HGG affirms all patients with HGG should be considered for referral to specialist palliative care, based on need rather than prognosis [23]. Interestingly, our results suggest palliative care is more integrated in regional/rural settings.

Throughout the OCP, the GP (primary care/family doctor) features in many steps from diagnosis to end-of-life care [10]. Our findings highlighted the low level of primary care integration with subspecialty team care; however, opinions regarding GP integration with patient care were not explored in depth. While patients were usually directed to their GP for care of pre-existing conditions, HPs did not routinely recommend GPs take a major role in non-oncological issues associated with the cancer diagnosis. This differs from oncology models which recommend GPs take a major role in supportive care assessments and referral to services [23].

A proportion of HPs perceived caring for carers, including recommending GP engagement, was not part of their role. At least 25% of HPs did not know or never advised carers to present to a GP. The disconnect between carers and the patient’s GP has been reported with variation seen for GPs perceived scope of practice, knowledge, and skills [38].

A recent RCT testing an intervention to improve continuity of care between oncology and family practice teams, reported better continuity of information and management [39]. GPs could provide an important service for HGG patients and their carers, particularly for those managing difficult or changing symptoms, for financial concerns, and links to appropriate services [40].

### Limitations

COGNO was the sole organisation involved in survey distribution and the number of participants was small, limiting representativeness of responses. However, the small number was not unexpected given that brain tumours are rare and sites may have nominated one HP to complete the survey. As the survey focused on supportive care not all members were expected to respond. Previous HP surveys have achieved 15–20% response rates [41].

Many of the responses received for this survey were from clinicians based in public hospitals in the metropolitan area and 81% of these were tertiary referral cancer centres. This is consistent with the Australian population density being centred in metropolitan cities with some spread to regional coastal areas and smaller numbers in rural areas [42]. Additionally, data shows that more brain cancers were diagnosed in major cities compared to regional and remote areas [43]. Finally, a higher response rate from participants in tertiary referral cancer centres was expected with many neuro-oncology specialists working in multidisciplinary teams in tertiary cancer centres in metropolitan areas. In Australia, there is limited specialist neuro-oncology care available or provided in regional areas and in the private sector.

Our interpretation of the data was limited by missing data due to attrition and question changes to address pilot feedback. Due to small sample sizes, exact probabilities (FET tests) rather than approximate tests were used; however, there were only n = 6 rural/regional responses therefore these findings should be interpreted cautiously. The survey did not include questions about MDT teleconferencing which has become an important component of MDT delivery since the COVID-19 pandemic. Additionally, questions about resources for patients and carers from non-English speaking backgrounds were not included.

### Recommendations

Based on the OCP [10], international guidelines for management of brain tumours [12], input from the clinical members in our team and our results, key gaps in supportive care for patients with HGG and their carers could be addressed by:

- Increasing neuro-oncology MDTs involvement of supportive care and psychosocial support staff
- Improving access to Cancer Care Coordinators and specialist nurses
- More consistent referral and access to mental health services for patients and carers regardless of location
- Early referral to allied health services
- Referral to rehabilitation services to support patient function and quality of life
- Early involvement of palliative care services
- Recognising and facilitating the GP’s role in supporting patients and carers in the community.

In regional and/or rural settings it may not be possible to provide on-site access to all of the support required to patients diagnosed with cancer and their carers. Appropriate referral and access to telehealth services is likely to be beneficial in ensuring patients and carers can access the timely support they require regardless of geographic location.

## Conclusion

The survey revealed that while many key services are accessible to patients diagnosed with HGG in Australia, improvements are needed. Integration of psychosocial support into routine care appears to be a critical gap even in tertiary cancer centres based in metropolitan areas. There is also a need to advocate for better integration of specialist allied health and primary care providers to improve care and patient and carer quality of life.

## Supplementary Information

Below is the link to the electronic supplementary material.

Supplementary file1 (PDF 1196 kb)

Supplementary file2 (DOCX 129 kb)

Supplementary file3 (DOCX 13 kb)

Supplementary file4 (DOCX 32 kb)

## Data Availability

The datasets generated during and/or analysed during the current study are available from the corresponding author on reasonable request.
