# Supplementary material for: Supportive care of patients diagnosed with high grade glioma and their carers in Australia
Source: J Neurooncol. 2022 Apr 9;157(3):475–85. doi: 10.1007/s11060-022-03991-z (PMC8994178; doi:10.1007/s11060-022-03991-z)
Supplement: Supplementary file 1 — Supplementary file1 (PDF 1196 kb) [file 11060_2022_3991_MOESM1_ESM.pdf]

Demo

# Survey of health professionals to determine current usual care provided to adult patients diagnosed with High Grade Glioma and their Carers

**All questions relate to your primary site of neuro-oncology practice, i.e. the site at which you would see the largest number of patients with high grade glioma.**

## Demographics

### In which State or territory is your workplace based?

- ◯ NSW
- ◯ VIC
- ◯ QLD
- ◯ SA
- ◯ WA
- ◯ ACT
- ◯ TAS
- ◯ NT

### What is the postcode of your primary neuro-oncology workplace?

### Please indicate the nature of your primary neuro-oncology workplace location?

- ◯ Major city
- ◯ Regional/Rural
- ◯ Remote

### Is your main interaction with neuro-oncology patients in...?

- ◯ Hospital Public
- ◯ Hospital Private
- ◯ Private Practice
- ◯ Both Public and Private
- ◯ Other – Please Specify

### Type of clinical setting

- ◯ Tertiary referral cancer centre
- ◯ District/local hospital
- ◯ Non-inpatient cancer treatment centre
- ◯ Non-hospital based practice
- ◯ Other – please Specify

### What is your discipline?

- ◯ Medical oncologist

- ◯ Neuro-oncologist
- ◯ Radiation oncologist
- ◯ Neurosurgeon
- ◯ Nurse or care coordinator
- ◯ Allied health (Physiotherapist, Occupational Therapist, Social Worker)
- ◯ Psychiatrist, clinical psychologist or psycho-oncologist
- ◯ Palliative care physician
- ◯ Rehabilitation physician
- ◯ Other

**What is your highest level of training?**

**Years practising in your discipline?**

**Years practising in your current position?**

**On average, approximately how many new patients with high grade glioma (HGG) does your primary site of neuro-oncology practice see per year?**

**What is your age?**

**What is your gender?**

- ◯ Male
- ◯ Female
- ◯ Other (please state
- ◯ Prefer not to answer

**MDT-Care**

## Multidisciplinary care of patients with HGG

**At your site do you have a neuro-oncology multidisciplinary team meeting?**

- ◯ Yes
- ◯ No

**If YES, What percentage of patients newly diagnosed with high grade glioma are discussed at a multidisciplinary team meeting?**

**If YES, how frequently does the multidisciplinary team formally meet?**

- ◯ Weekly
- ◯ Fortnightly
- ◯ Monthly
- ◯ Other frequency. Please state

**If YES, which disciplines attend most multidisciplinary team meetings (either in person or remotely)?**

- ☐ Neurosurgeon
- ☐ Radiation oncologist
- ☐ Medical Oncologist
- ☐ Neuro-oncologist
- ☐ Radiologist
- ☐ Pathologist
- ☐ Neuropathologist
- ☐ Neurosurgery nurse
- ☐ Care coordinator
- ☐ Medical oncology nurse
- ☐ Pharmacist
- ☐ Radiation oncology nurse
- ☐ Social worker
- ☐ Physiotherapist
- ☐ Occupational therapist
- ☐ Palliative care specialist
- ☐ Palliative care nurse
- ☐ Clinical trials coordinator
- ☐ Dietician
- ☐ General Practitioner
- ☐ Neurologist
- ☐ Neuropsychologist
- ☐ Psychiatrist
- ☐ Nuclear medicine physician
- ☐ Rehabilitation physician
- ☐ Speech therapist
- ☐ Other

**At your site can you refer patients diagnosed with HGG to a...**

| | Yes (on site) | External or private provider | This service is not available at my site | Unsure, I do not refer to this service |
|---|---|---|---|---|
| Cancer care coordinator/nurse navigator | ◯ | ◯ | ◯ | ◯ |
| Nurse practitioner | ◯ | ◯ | ◯ | ◯ |
| Neuro surgery nurse | ◯ | ◯ | ◯ | ◯ |
| Radiation oncology nurse | ◯ | ◯ | ◯ | ◯ |
| Oral chemotherapy nurse | ◯ | ◯ | ◯ | ◯ |
| Oral chemotherapy/oncology pharmacist | ◯ | ◯ | ◯ | ◯ |
| Seizure or epilepsy nurse | ◯ | ◯ | ◯ | ◯ |

| | Yes (on site) | External or private provider | This service is not available at my site | Unsure, I do not refer to this service |
|---|---|---|---|---|
| Social worker or welfare officer | ○ | ○ | ○ | ○ |
| Dietitian | ○ | ○ | ○ | ○ |
| Neuro-psychologist for cognitive function testing (neuropsychological/neuropsychiatric testing) | ○ | ○ | ○ | ○ |
| General psychologist | ○ | ○ | ○ | ○ |
| Oncology psychologist | ○ | ○ | ○ | ○ |
| Counsellor, including telephone service | ○ | ○ | ○ | ○ |
| Psychiatrist | ○ | ○ | ○ | ○ |
| Occupational therapist | ○ | ○ | ○ | ○ |
| Physiotherapist | ○ | ○ | ○ | ○ |
| Hospital based palliative care specialist or inpatient palliative care | ○ | ○ | ○ | ○ |
| Domiciliary palliative care service (may or may not be linked with hospital) | ○ | ○ | ○ | ○ |
| Rehabilitation physician | ○ | ○ | ○ | ○ |
| Speech therapist | ○ | ○ | ○ | ○ |
| Exercise physiologist | ○ | ○ | ○ | ○ |
| Other support services ______ | ○ | ○ | ○ | ○ |

**The table below lists the services you indicated were available at your site. Which scenario most closely corresponds to the use of this service for patients with HGG at your site?**

**As a guide for your answers please consider:**

- **Always/routinely to be 95% or more**
- **Most to be 70-95%**
- **About half to be 30-70%**
- **Some to be 5-30%**
- **Very few to be less than 5%**

| | **Of the patients with HGG at my site** | | | | | **Or** |
|---|---|---|---|---|---|---|
| | All are routinely offered/referred to this service | Most are offered/referred to this service | About half are offered/referred to this service | Some are offered/referred to this service | Very few are offered/referred to this service | This service is available but I do not know how often it is used |
| Cancer care coordinator/nurse navigator | ○ | ○ | ○ | ○ | ○ | ☐ |
| Nurse practitioner | ○ | ○ | ○ | ○ | ○ | ☐ |
| Neuro surgery nurse | ○ | ○ | ○ | ○ | ○ | ☐ |
| Radiation oncology nurse | ○ | ○ | ○ | ○ | ○ | ☐ |
| Social worker or welfare officer | ○ | ○ | ○ | ○ | ○ | ☐ |
| Dietitian | ○ | ○ | ○ | ○ | ○ | ☐ |
| Neuro-psychologist for cognitive function testing (neuropsychological/neuropsychiatric testing) | ○ | ○ | ○ | ○ | ○ | ☐ |
| General psychologist | ○ | ○ | ○ | ○ | ○ | ☐ |
| Oncology psychologist | ○ | ○ | ○ | ○ | ○ | ☐ |
| Counsellor, including telephone service | ○ | ○ | ○ | ○ | ○ | ☐ |
| Psychiatrist | ○ | ○ | ○ | ○ | ○ | ☐ |
| Occupational therapist | ○ | ○ | ○ | ○ | ○ | ☐ |
| Physiotherapist | ○ | ○ | ○ | ○ | ○ | ☐ |

| | Of the patients with HGG at my site | | | | | Or |
|---|---|---|---|---|---|---|
| | All are routinely offered/referred to this service | Most are offered/referred to this service | About half are offered/referred to this service | Some are offered/referred to this service | Very few are offered/referred to this service | This service is available but I do not know how often it is used |
| Hospital based palliative care specialist or inpatient palliative care | ○ | ○ | ○ | ○ | ○ | ☐ |
| Domiciliary palliative care service (may or may not be linked with hospital) | ○ | ○ | ○ | ○ | ○ | ☐ |
| Rehabilitation physician | ○ | ○ | ○ | ○ | ○ | ☐ |
| Speech therapist | ○ | ○ | ○ | ○ | ○ | ☐ |
| Exercise physiologist | ○ | ○ | ○ | ○ | ○ | ☐ |
| Other support services | ○ | ○ | ○ | ○ | ○ | ☐ |

**The table below lists a service you indicated was available at your site. Which scenario most closely corresponds to the use of this service for patients at your site diagnosed with HGG and who have had seizures?**

| | Of patients at my site, with HGG and who have had seizures | | | | | Or |
|---|---|---|---|---|---|---|
| | All are routinely offered/referred to this service | Most are offered/referred to this service | About half are offered/referred to this service | Some are offered/referred to this service | Very few are offered/referred to this service | This service is available but I do not know how often it is used |
| Seizure or epilepsy nurse | ○ | ○ | ○ | ○ | ○ | ☐ |

**The table below lists one or more services you indicated were available at your site. Which scenario most closely corresponds to the use of this service for patients at your site diagnosed with HGG and who are starting oral chemotherapy?**

| | Of patients at my site with HGG who are starting oral chemotherapy | | | | | Or |
|---|---|---|---|---|---|---|
| | All are routinely offered/referred to this service | Most are offered/referred to this service | About half are offered/referred to this service | Some are offered/referred to this service | Very few are offered/referred to this service | This service is available but I do not know how often it is used |
| Oral chemotherapy nurse | ○ | ○ | ○ | ○ | ○ | ☐ |
| Oral chemotherapy/oncology pharmacist | ○ | ○ | ○ | ○ | ○ | ☐ |

**For which health issues are patients diagnosed with HGG specifically advised to seek care from their <u>general practitioner</u>?**

- ☐ Pre-existing chronic conditions or comorbidities
- ☐ Psychological support or psychiatric issues
- ☐ Rehabilitation and physical function
- ☐ Social issues (e.g. ACROD permit, insurance, driving)
- ☐ Side effects of their cancer treatment (e.g. nausea, hypertension, allergic reactions)
- ☐ Complications of their cancer (e.g. deep vein thrombosis)
- ☐ Symptoms of their cancer (e.g. headache, seizure management)

☐ Other

☐ Not applicable

**What proportion (%) of patients receiving treatment for HGG at your site are given the following types of information:**

**As a guide for your answers please consider:**

- **Always/routinely to be 95% or more**
- **Most to be 70-95%**
- **About half to be 30-70%**
- **Some to be 5-30%**
- **Very few to be less than 5%**

| | **Of patients with HGG at my site** | | | | | **Or** |
|---|---|---|---|---|---|---|
| | All are routinely given this information | Most are given this information | About half are given this information | Some are given this information | Very few are given this information | Don't know |
| Written information specifically developed by your site | ○ | ○ | ○ | ○ | ○ | ☐ |
| Written information developed at state or national level (e.g. Cancer Council, eVIQ information) | ○ | ○ | ○ | ○ | ○ | ☐ |
| Written information developed by a pharmaceutical company or device manufacturer | ○ | ○ | ○ | ○ | ○ | ☐ |
| Group information sessions run by your site | ○ | ○ | ○ | ○ | ○ | ☐ |
| Online information specifically developed by your site | ○ | ○ | ○ | ○ | ○ | ☐ |
| Information about online resources not developed by your site | ○ | ○ | ○ | ○ | ○ | ☐ |

**At your site can you refer patients diagnosed with HGG to a...**

| | Yes (on site) | External or private provider | This service is not available at my site | Unsure, I do not refer to this service |
|---|---|---|---|---|
| Pastoral care or a person who provides spiritual support | ○ | ○ | ○ | ○ |
| Complementary therapy service provider | ○ | ○ | ○ | ○ |
| Fitness to drive assessment | ○ | ○ | ○ | ○ |
| Support with legal issues (advance care planning, power of attorney, wills) | ○ | ○ | ○ | ○ |
| Support group | ○ | ○ | ○ | ○ |

**The table below lists the services you indicated were available at your site. Which scenario most closely corresponds to the use of this service for patients with HGG at your site?**

**As a guide for your answers please consider:**

- **Always/routinely to be 95% or more**
- **Most to be 70-95%**
- **About half to be 30-70%**
- **Some to be 5-30%**
- **Very few to be less than 5%**

| | Of the patients with HGG at my site | | | | | Or |
|---|---|---|---|---|---|---|
| | All are routinely offered/referred to this service | Most are offered/referred to this service | About half are offered/referred to this service | Some are offered/referred to this service | Very few are offered/referred to this service | This service is available but I do not know how often it is used |
| Pastoral care or a person who provides spiritual support | ○ | ○ | ○ | ○ | ○ | ☐ |
| Complementary therapy service provider | ○ | ○ | ○ | ○ | ○ | ☐ |
| Fitness to drive assessment | ○ | ○ | ○ | ○ | ○ | ☐ |
| Support with legal issues (advance care planning, power of attorney, wills) | ○ | ○ | ○ | ○ | ○ | ☐ |
| Support group | ○ | ○ | ○ | ○ | ○ | ☐ |

**Is there a support group specifically for HGG patients or brain cancer patients?**

○ Yes, HGG patients (+/- carers)

○ Yes, brain cancer patients (+/- carers)

○ Yes, mixed cancer patients (+/- carers)

○ No

**Carers**

## Multidisciplinary care of carers of patients with HGG (e.g. family members or informal supporters)

**At your site can you refer a carer of a HGG patient for support from a...**

| | Yes (on site) | External or private provider | This service is not available at my site | Unsure, I do not refer to this service |
|---|---|---|---|---|
| Cancer care coordinator/nurse navigator | ○ | ○ | ○ | ○ |
| Nurse practitioner | ○ | ○ | ○ | ○ |
| General psychologist | ○ | ○ | ○ | ○ |
| Social worker or welfare officer | ○ | ○ | ○ | ○ |
| Support group | ○ | ○ | ○ | ○ |
| Other, please specify | ○ | ○ | ○ | ○ |

**The table below lists the services you indicated were available at your site. Which scenario most closely corresponds to the use of this service for carers of patients with HGG at your site?**

**As a guide for your answers please consider:**

- **Always/routinely to be 95% or more**
- **Most to be 70-95%**
- **About half to be 30-70%**
- **Some to be 5-30%**
- **Very few to be less than 5%**

| | Of the carers of patients with HGG at my site | | | | | Or |
|---|---|---|---|---|---|---|
| | All are routinely offered/referred to this service | Most are offered/referred to this service | About half are offered/referred to this service | Some are offered/referred to this service | Very few are offered/referred to this service | This service is available but I do not know how often it is used |
| Cancer care coordinator/nurse navigator | ○ | ○ | ○ | ○ | ○ | ☐ |
| Nurse practitioner | ○ | ○ | ○ | ○ | ○ | ☐ |
| General psychologist | ○ | ○ | ○ | ○ | ○ | ☐ |
| Social worker or welfare officer | ○ | ○ | ○ | ○ | ○ | ☐ |
| Support group | ○ | ○ | ○ | ○ | ○ | ☐ |

**At your site can you refer a carer of a HGG patient for support from a support group?**

- ☐ Yes, group for carers of HGG patients
- ☐ Yes, group for carers of brain cancer patients
- ☐ Yes, group for HGG patients and carers
- ☐ Yes, group for brain cancer patients and carers
- ☐ Yes, group for carers of cancer patients (any diagnosis)
- ☐ Yes, group for cancer patients (any diagnosis) and carers
- ☐ No

**At each of the following time points, what proportion of carers of HGG patients are advised to present to their general practitioner?**

**As a guide for your answers please consider:**

- **Always/routinely to be 95% or more**
- **Most to be 70-95%**
- **About half to be 30-70%**
- **Sometimes to be 5-30%**
- **Very few to be less than 5%**

| | Of carers of patients with HGG at my site | | | | | Or | |
|---|---|---|---|---|---|---|---|
| | All are routinely advised to visit their GP | Most are advised to visit their GP | About half are advised to visit their GP | Some are advised to visit their GP | Very few are advised to visit their GP | Never advised to present to GP as carer is not receiving medical advice | Don't know |
| Advised on diagnosis of their loved one | ○ | ○ | ○ | ○ | ○ | ○ | ○ |
| Advised during inpatient hospitalisation/s of their loved one | ○ | ○ | ○ | ○ | ○ | ○ | ○ |
| Advised during outpatient care of their loved one | ○ | ○ | ○ | ○ | ○ | ○ | ○ |

| | Of carers of patients with HGG at my site | | | | | Or | |
|---|---|---|---|---|---|---|---|
| | All are routinely advised to visit their GP | Most are advised to visit their GP | About half are advised to visit their GP | Some are advised to visit their GP | Very few are advised to visit their GP | Never advised to present to GP as carer is not receiving medical advice | Don't know |
| Advised as needed at any point during care of their loved one | ○ | ○ | ○ | ○ | ○ | ○ | ○ |

**Please describe the main reasons why a carer might be advised to present to their GP**

**What other support is available for carers of HGG patients at your site?**

**Is there anything else you would like to share about current usual care provided to patients diagnosed with High Grade Glioma and their Carers?**
