# Supplementary material for: Supportive care of patients diagnosed with high grade glioma and their carers in Australia
Source: J Neurooncol. 2022 Apr 9;157(3):475–85. doi: 10.1007/s11060-022-03991-z (PMC8994178; doi:10.1007/s11060-022-03991-z)
Supplement: Supplementary file 2 — Supplementary file2 (DOCX 129 kb) [file 11060_2022_3991_MOESM2_ESM.docx]

Supportive care of patients diagnosed with High Grade Glioma and their carers in Australia

Georgia KB Halkett^1^, Melissa Berg^1,2^, Davina Daudu^1, 3^, Haryana M Dhillon ^4,5^, Eng-Siew Koh^6-8^, Tamara Ownsworth^9^, Elizabeth Lobb^10,11,12^, Jane Phillips^13^, Danette Langbecker^14^, Meera Agar^12^, Elizabeth Hovey^6,15,16^, Rachael Moorin^17,18^, Anna K. Nowak^3, 19^

^1^ Curtin School of Nursing, Faculty of Health Sciences, Curtin University, Bentley, Western Australia

^2^ School of Nursing and Midwifery, The University of Notre Dame Australia, Fremantle, Western Australia, Australia

^3^Medical School, University of Western Australia, Nedlands, Western Australia.

^4^Psycho-Oncology Cooperative Research Group, School of Psychology, Faculty of Science, University of Sydney, NSW

^5^Centre for Medical Psychology & Evidence-based Decision-making, School of Psychology, Faculty of Science, University of Sydney, NSW

^6^Faculty of Medicine, University of New South Wales, NSW

^7^Liverpool and Macarthur Cancer Therapy Centres, Liverpool, Australia

^8^Ingham Institute for Applied Medical Research, Liverpool, Australia

^9^School of Applied Psychology & The Hopkins Centre, Menzies Health Institute Queensland
Griffith University, Queensland

^10^Calvary Health Care Kogarah, Sydney, New South Wales

^11^School of Medicine, The University of Notre Dame, Sydney, New South Wales.

^12^Faculty of Health, University of Technology, Ultimo, New South Wales

^13^ School of Nursing, Faculty of Health, Queensland University of Technology

^14^ Centre for Online Health and Centre for Health Services Research, The University of Queensland, Brisbane, Australia.

^15^ Faculty of Medicine and Health, University of Sydney, Sydney, Australia.

^16^ Nelune Comprehensive Cancer Centre, Randwick, Australia.

^17^ Curtin School of Population Health, Faculty of Health Sciences, Curtin University, Bentley, WA

^18^ School of Population and Global Health, University of Western Australia, Nedlands, Western Australia.

^19^ Department of Medical Oncology, Sir Charles Gairdner Hospital, Nedlands, Western Australia.

# Abstract (250 words)

Purpose: This study aimed to: determine the supportive care available for Australian patients with High Grade Glioma (HGG) and their carers; identify service gaps; and inform changes needed to implement guidelines and Optimal Care Pathways.

Methods: This cross-sectional online survey recruited multidisciplinary health professionals (HPs) who were members of the Cooperative Trials Group for Neuro-Oncology involved in management of patients diagnosed with HGG in Australian hospitals. Descriptive statistics were calculated. Fisher's exact test was used to explore differences between groups.

Results: 42 complete responses were received. A majority of MDT meetings were attended by: neurosurgeon, radiation oncologist, medical oncologist, radiologist, and care coordinator. Less than 10% reported attendance by palliative care nurse; physiotherapist; neuropsychologist; speech therapist. Most could access referral pathways to a cancer care coordinator(76%), neuropsychologist(78%), radiation oncology nurse(77%), or psycho-oncologist(73%), palliative care(93-100%) and mental health professionals(60-85%) . However, few routinely referred to an exercise physiologist(10%), rehabilitation physician(22%), dietitian(22%) or speech therapist(28%).Similarly, routine referrals to specialist mental health services were not standard practice. Nearly all HPs(94%) reported HGG patients were advised to present to their GP for pre-existing conditions/comorbidities; however, most HPs took responsibility(≤36% referred to GP) for social issues, mental health, symptoms, cancer complications, and treatment side-effects.

Conclusions: While certain services are accessible to HGG patients nationally, improvements are needed. Psychosocial support, specialist allied health, and primary care providers are not yet routinely integrated into the care of HGG patients and their carers despite these services being considered essential in clinical practice guidelines and optimal care pathways.

# Introduction

Internationally, more than 330,000 cases of Central Nervous System tumours are diagnosed annually with an age-standardised incidence rate of 4.63 per 100,000[1]. Five-year relative survival rates are poor, with 22%-24% survival for malignant brain and other CNS tumours in Australia and the United States (US)[2, 3]. In this context, we use the term ‘high grade glioma’ (HGG) to encompass Glioblastoma IDH wild type, Astrocytoma IDH mutant (grade 3,4), and oligodendroglioma IDH-mutant 1p/19q co-deleted (grade 3)[4]. Standard treatments include surgery, radiation therapy, and chemotherapy.

Adults diagnosed with HGG experience functional and neurological deficits, and behavioural and personality changes[5]. Symptom severity ranges from minimal disruption to everyday activities to the patient being fully care-dependent[6]. Consequently, people diagnosed with HGG and their carers experience high levels of distress and have significant unmet supportive care needs[6-8]. Patients and their carers require timely access to support and evidence-based information to manage their disease and its impact[9].

Cancer Care pathways are established to ensure people receive quality cancer care[10]. The Australian Optimal Care Pathway (OCP) for HGG details seven principles of care and a seven-step care pathway encompassing prevention and early detection through to end of life. Supportive care supplements clinical treatment and addresses issues emerging from the cancer diagnosis and treatment. It comprises services, information and resources which meet the individual's physical, psychological, social, information, and spiritual needs[10]. The OCP pathway highlights the diversity of supportive care needs and importance of access to appropriate supportive care throughout the disease trajectory[10].

The Australian National Brain Cancer Audit concluded that care outcomes will improve if patients and carers have early access to care coordination, rehabilitation and survivorship support services[11]. Multidisciplinary teams and communication between team members and with patients and carers facilitate optimal care and ensure patients receive timely and appropriate management [11]. Similarly, international guidelines highlight the importance of providing access to multidisciplinary care, rehabilitation, psychosocial, and allied health support[12]. No previous research has examined the nature of support services for people with HGG and their carers. This study aimed to: determine the supportive care available for patients with HGG and their carers, identify gaps in services and inform best-practice implementation of guidelines and OCPs for this group.

# Methods

## Study design and setting

A cross-sectional online survey documenting supportive care available at clinical sites in Australia was conducted November-December 2018. Ethics approval was granted by Curtin University (HRE2018-0706). The checklist for reporting results of internet e-surveys guided this report[13].

## Recruitment

Multidisciplinary health professionals (HPs) who were members of the Cooperative Trials Group for Neuro-Oncology (COGNO), and involved in management of patients diagnosed with HGG in Australian hospitals were invited to participate.

In 2018 COGNO had approximately 662 members [14]. Surveys were sent to members in clinical disciplines: medical/neuro/radiation-oncology, neurosurgery, nursing, trainees/registrars, allied health (e.g. physiotherapist, occupational therapist, social worker), palliative care, rehabilitation, and psychology. Responses were monitored and reminders targeted to HPs in sites and states with no/low responses. Anecdotal feedback suggested some sites nominated one person to respond or discussed responses as a team as the survey focused on describing what was available to patients at their site as a whole rather than individual experiences in making referrals. Consequently, it was not possible to calculate the response rate.

## Instrument

Questionnaire development was guided by the OCP for people with HGG[15]. The questionnaire was reviewed by representatives from the COGNO Scientific Advisory Committee and Consumer Advisory Panel and piloted by a subset of the sample (n=11) (Supplement 1 details modifications).

The final questionnaire comprised 38 questions in three sections: 1) socio-demographics; 2) multidisciplinary care; and, 3) multidisciplinary care of carers (Supplement 2). A final question invited open-ended comments about usual care.

### Socio-demographics

Twelve socio-demographic questions detailed practice location, clinical setting, professional discipline, training, years practicing, new patients treated at site/year, age, and gender.

### Multidisciplinary care of patients with HGG

This section included 13 questions on: existence and frequency of neuro-oncology multidisciplinary team (MDT) meetings and attending disciplines; hospital or external supportive care services and proportion of patients referred; advice about when to present to general practitioners (GPs); type and frequency of information offered. Supportive care services were grouped by domains aligning with the OCP[15].

### Multidisciplinary care of carers of patients with HGG

Six questions documented: supportive care services available to carers, proportion of carers referred; advice regarding when to present to GPs; and other types of support available (open ended response).

## Procedure

Australian COGNO members from eligible disciplines received an email invitation from COGNO to complete the anonymous online questionnaire and forward it to a colleague. The email provided a link to the information sheet, consent form and questionnaire (Qualtrics, Provo, UT). Investigators also disseminated the survey link through their professional networks.

Participants consented online prior to commencing the survey. Two reminders were sent. The pilot survey was distributed on 8 November 2018 and 13 responses received. The survey was modified after feedback (Supplement 1) and redistributed on 29 November 2018 and remained open for 5.6 weeks. Data collected during the pilot was included. For two questions with modified responses, pilot data were manually transformed. Pilot data collected on the proportion of patients referred to services could not be transformed, resulting in some missing data. Two researchers (GH, MB) identified duplicates using demographic responses and for agreed duplicate pairs, earlier responses were removed.

## Data analysis

Analysis was completed using IBM SPSS Version 27. Incomplete surveys were included, with missing data for each section identified. Descriptive statistics were calculated. The OCP states most HGG patients will require specialised supportive care, therefore, we expected at least half of patients would be referred to a particular service [11]. Fisher's Exact Tests (FET)[16] were used to explore differences between groups in cross-tabulations The degrees of freedom (df) are shown in significant tests where df>1. A *p* value < 0.05 was deemed statistically significant. Content analysis was used to compile free-text responses.

# Results

Consent was obtained from 55 HPs (n=13 pilot; n=42 final); six answered no questions and seven responses were duplicates, leaving 42 responses (n=5 pilot; n=37 final). Respondents took < 15 minutes to complete the survey (IQ_25_=7.67 minutes; IQ_75_=14.67 minutes).

Table 1 summarises HP socio-demographic data.

## Neuro-oncology multidisciplinary team meetings

Table 2 summarises data about neuro-oncology MDT meetings. Almost all HPs reported their site held neuro-oncology MDT meetings (95%) and most discussed all patients newly-diagnosed with HGG (62%). The following specialist HPs were most frequently reported as attending MDT meetings: neurosurgeon (100%), radiation oncologist (95%), medical oncologist (95%), radiologist (87%), and care coordinator (77%). A neuropathologist (64%) or pathologist (51%) were less likely to attend. Minimal MDT attendance was reported for: palliative care nurse (8%); nuclear medicine physician (8%); physiotherapist (5%); neuropsychologist (5%); and, speech therapist (3%).

A significantly greater proportion of metropolitan HPs held neuro-oncology MDT meetings at their site (100%) compared with HPs working in a regional/rural location (67%; p=0.017). A significantly greater proportion of HPs working in a tertiary cancer centre had a neuro-oncology MDT meeting (100%) compared with those in a district/local hospital (75%) or non-inpatient cancer treatment centre (75%; df=2, p=0.033).

## Multidisciplinary care of patients diagnosed with HGG

### Physical, psychological and social domains

Supportive care services addressing needs in the physical, psychological and social domains are summarised in Table 3. At their site, a majority of HPs (85%-100%) could refer patients to a physiotherapist, hospital-based palliative care, speech therapist, dietitian, social worker, rehabilitation physician, occupational therapist, domiciliary palliative care service, psychiatrist, or general psychologist. Between 72-78% could refer patients to a cancer care coordinator, neuropsychologist, radiation oncology nurse, or psycho-oncologist. Two-thirds could refer patients starting oral chemotherapy to a pharmacist and 54% could refer to an oral chemotherapy nurse while most HPs (60%-85%) could refer patients to a psychology, psychiatry, or counselling service provider.

More metropolitan HPs could refer patients to a neuropsychologist (metropolitan=85%; regional/rural=33%; p=0.016). Similarly, more HPs who worked in a tertiary cancer centre could refer to a neuropsychologist (84%) or rehabilitation physician (97%) compared with HPs from district/local hospitals (25%; df=2, p=0.033; 50%, df=2, p=0.042 respectively).

At sites with each service available, the proportion of HGG patients referred to supportive care services is shown in Figure 1. Disciplines with infrequent referrals included exercise physiology, rehabilitation physician, speech therapist, or dietitian. Approximately half of HPs stated some/very few patients were referred to an oral chemotherapy/oncology pharmacist. Most HPs stated some/very few patients were referred to mental health services: counsellor (including telephone service), general psychologist, neuropsychologist, psychiatrist, or psycho-oncologist.

Compared to metropolitan HPs, more HPs in a regional/rural location stated at least half of HGG patients were referred to: oral chemotherapy nurse (regional/rural=100%; metropolitan=23%; p=0.007); an occupational therapist (regional/rural=100%; metropolitan=48%; p=0.028); a physiotherapist (regional/rural=100%; metropolitan=46%; p=0.024); or to hospital-based palliative care (regional/rural=100%; metropolitan=43%; p=0.020).

### Support groups, spiritual, practical and information domains

Spiritual and practical supportive services and support groups were available to most patients (Table 4).

A significantly greater proportion of HPs from a tertiary cancer centre could refer patients for a fitness-to-drive assessment compared with a district/local hospital (86% vs. 25%; df=2, p=0.029).

At sites where spiritual or practical supportive care was available, the proportion of patients referred is shown in Supplement 3(Figure 1). Supplement 3(Table 1) summarises the proportion of patients newly diagnosed with HGG who are given various sources of information.

### Health issues referred to General Practitioners (GP)

Nearly all HPs (94%) reported HGG patients were advised to present to their GP for pre-existing conditions/comorbidities. Fewer advised patients to see their GP for social issues (36%), mental health (19%), symptoms (11%), cancer complications (11%), and treatment side-effects (3%) (Supplement 4).

## Care of carers

Thirty-five HPs provided data about the multidisciplinary care of carers of patients with HGG. Availability of specialist nursing, psychological, and social supportive services for carers and the proportion of carers referred are shown in Supplement 5 (Table 1 and Figure 1). Most HPs (62%-83%) could refer carers to a social worker/welfare officer, care coordinator/nurse navigator, or psychologist.

Of HPs who answered the question (n=32), approximately half could refer a HGG patient carer to any support group (Supplement 5–Table 2). Supplement 5-Table 3 shows the proportion of carers of HGG patients advised to present to their general practitioner at different timepoints during treatment. At least 25% indicated they either did not know or that carers were never advised to see a GP about their 'loved one'. The main reasons that carers might be advised to present to their GP included: psychological support (n=20), carers health (n=5), and assistance to complete paperwork for financial support (n=1).

# Discussion

This study documented the supportive care available and routinely utilised for patients with HGG and their carers. Our survey specifically investigated key recommendations within the OCP, which outlines the nationally agreed best practice for HGG[10].

According to best international evidence, MDT meetings are critical in managing brain tumours[12] and newly-diagnosed patients should have a MDT recommended plan within two weeks of diagnosis or before surgery[10]. Almost all responding sites reported regular neuro-oncology MDT meetings at which most new patients were discussed. Meetings appear most focused on initial treatment planning. Few HPs reported care coordinators, neurosurgery nurses and social workers attended, despite their designation as core members of a neuro-oncology MDT[10]. Professionals from disciplines providing supportive care and psychosocial support rarely attended. Consequently, psychosocial, allied health, rehabilitation, and supportive care needs may be under-addressed [10].

Cancer Care Coordinators and specialist nurses play a key role in supporting this group[10, 17]. Access to specialist nursing care varied, with a quarter of sites lacking a cancer care coordinator. However, when available, nearly all patients were referred. We identified important potential service gaps including limited access to neurosurgery, oral chemotherapy, and seizure or epilepsy nurses. Despite the prevalence of seizures in people with HGG[17-19], only 15% reported access to a seizure/epilepsy nurse. When available, half of HGG patients were referred, highlighting service relevance. In other settings, care provided by seizure/epilepsy nurses may be associated with reductions in seizure-related Emergency Department (ED) and GP presentations[20, 21].

Best evidence highlights the importance of screening for supportive care needs and providing access to relevant support services[10, 22]. Screening for supportive care needs should begin at presentation and occur regularly[23]. Although we did not capture timing of referral, we documented service availability and the proportion of patients and carers referred to each along the care pathway.

Only some patients were referred to mental health services, with some only available through external providers, presenting an access barrier. Australian clinical practice guidelines and OCPs indicate early consistent psychosocial care of brain cancer patients and their carers is critical[10, 18, 24, 25]. Most cognitive rehabilitation intervention programs reported improvements in patients' cognitive test-performance[26] and could be considered for inclusion in psychosocial care of HGG patients. Future research will provide greater understanding of psychological management of people with brain tumour and their carers, including screening for psychological distress and cognitive deficits, and how these issues are managed in practice. Improved access to psychological interventions through remote delivery is an important consideration. However, telehealth research indicates uptake and adherence are higher for interventions involving real-time interactions rather than self-guided interventions[27].

The OCP emphasises the importance of early allied health referral when required[10]. We found low referral to dietitians, who have a key role in the multidisciplinary care of neuro-oncology patients. Patients with brain tumours often experience weight gain resulting from corticosteroids and mobility limitations, and may benefit from dietitian consultations. We identified low referrals to speech therapists, despite the frequent occurrence of speech aberrations (eg. expressive and receptive dysphasia)[18] and increasing communication deficits over time[28]. Occasionally issues relating to swallowing emerge, which can be addressed by speech pathologists[29].

Rehabilitation plays an integral role in managing symptoms/complications of brain malignancies[30]. Although most HPs indicated availability of a rehabilitation physician, only 20% stated at least half their patients were referred. Patients with brain cancer who receive early intervention make comparable gains and report similar levels of satisfaction with post-surgical rehabilitation to those with stroke[31]. Accordingly, access to cognitive and physical rehabilitation is important to support management of functional loss and activity limitations[23, 32]. Exercise interventions for HGG patients can present challenges due to the burden of symptoms and other health-related commitments. However, they are perceived to be beneficial for patient health, a sense of control and social interaction, and carer respite[33].

Best evidence recommends palliative care is discussed early and, for teams not including a palliative care specialist, emphasises the importance of engaging primary care and community palliative care services[10, 34]. Despite the poor prognosis of HGG, involvement of palliative care services was relatively low. Few MDTs were attended by a palliative care clinician. Palliative care services were available at most sites; however, referral was not universal. Only a small proportion of HPs noted all patients were referred to palliative care. A systematic review of palliative care utilisation by glioblastoma patients identified advance care planning for up to half of patients, palliative care referrals and consultations for a third, and hospice referrals for most, with variable hospice use (38-86%)[35]. Although it is unclear from our survey whether low referral rates impacted the use of Advance Care Planning, such planning helps meet patients’ end of life preferences and reduces healthcare costs in patients with cognitive impairment or dementia[36]. The Australian National Palliative Care Standards states palliative care should be available to all patients with an active, progressive, or advanced disease[37] and the OCP for HGG affirms all patients with HGG should be considered for referral to specialist palliative care, based on need rather than prognosis[23]. Interestingly, our results suggest palliative care is more integrated in regional/rural settings.

Throughout the OCP, the GP (primary care/family doctor) features in many steps from diagnosis to end-of-life care [10]. Our findings highlighted the low level of primary care integration with subspecialty team care; however, opinions regarding GP integration with patient care were not explored in depth. While patients were usually directed to their GP for care of pre-existing conditions, HPs did not routinely recommend GPs taking a major role in non-oncological issues associated with the cancer diagnosis. This differs from oncology models which have recommended GPs take a major role in supportive care assessments and referral to services[23].

A proportion of HPs perceived caring for carers, including recommending GP engagement, was not part of their role. At least 25% of HPs did not know or never advised carers to present to a GP. The disconnect between carers and the patient’s GP has been reported with variation seen for GPs perceived scope of practice, knowledge, and skills[38].

A recent RCT testing an intervention to improve continuity of care between oncology and family practice teams, reported better continuity of information and management[39]. GPs could provide an important service for HGG patients and their carers, particularly for those managing difficult or changing symptoms, for financial concerns, and links to appropriate services[40].

## Limitations

COGNO was the sole organisation involved in survey distribution and the number of participants was small, limiting representativeness of responses. However, the small number was not unexpected given brain tumours are rare and sites may have nominated one HP to complete the survey. As the survey focused on supportive care not all members were expected to respond. Previous HP surveys have achieved 15-20% response rates[41].

Many of the responses received for this survey were from clinicians based in public hospitals in the metropolitan area and 81% of these were tertiary referral cancer centres. This is consistent with the Australian population density being centred in metropolitan cities with some spread to regional coastal areas and smaller numbers in rural areas[42]. Additionally, data shows that more brain cancers were diagnosed in major cities compared to regional and remote areas[43]. Finally, a higher response rate from participants in tertiary referral cancer centres was expected with many neuro-oncology specialists working in multidisciplinary teams in tertiary cancer centres in metropolitan areas. In Australia, there is limited specialist neuro-oncology care available or provided in regional areas and in the private sector.

Our interpretation of the data was limited by missing data due to attrition and question changes to address pilot feedback. Due to small sample sizes, exact probabilities (FET tests) rather than approximate tests were used; however, there were only n=6 rural/regional responses therefore these findings should be interpreted cautiously. The survey did not include questions about MDT teleconferencing which has become an important component of MDT delivery since the COVID-19 pandemic. Additionally, questions about resources for patients and carers from non-English speaking backgrounds were not included.

## Recommendations

Based on the OCP [10], international guidelines for management of brain tumours[12], input from the clinical members in our team and our results, key gaps in supportive care for patients with HGG and their carers could be addressed by:

- Increasing neuro-oncology MDTs involvement of supportive care and psychosocial support staff
- Improving access to Cancer Care Coordinators and specialist nurses
- More consistent referral and access to mental health services for patients and carers regardless of location
- Early referral to allied health services
- Referral to rehabilitation services to support patient function and quality of life
- Early involvement of palliative care services
- Recognising and facilitating the GP’s role in supporting patients and carers in the community.

In regional and/or rural settings it may not be possible to provide on-site access to all of the support required to patients diagnosed with cancer and their carers. Appropriate referral and access to telehealth services is likely to be beneficial in ensuring patients and carers can access the timely support they require regardless of geographic location.

# Conclusion

The survey revealed that while many key services are accessible to patients diagnosed with HGG in Australia, improvements are needed. Integration of psychosocial support into routine care appears to be a critical gap even in tertiary cancer centres based in metropolitan areas. There is also a need to advocate for better integration of specialist allied health and primary care providers to improve care and patient and carer quality of life.

# Acknowledgements

Davina Daudu was awarded a Cancer Council of WA Student Vacation Scholarship to contribute to data collection and analysis. Georgia Halkett is a recipient of a Cancer Council of WA Research Fellowship. We thank COGNO for assistance provided to recruit health professionals who participated in the study.

This study was part of a larger project funded by a PdCCRS Cancer Australia grant. Completion of this manuscript was supported by the BRAINS project funded by MRFF.

# References

1. GBD Brain and Other CNS Cancer Collaborators (2019) Global, regional, and national burden of brain and other CNS cancer, 1990-2016: a systematic analysis for the Global Burden of Disease Study 2016. Lancet Neurol 18: 376-393. doi:10.1016/S1474-4422(18)30468-X

2. AIHW (2019) Cancer in Australia 2019. AIHW, Canberra

3. Ostrom QT, Patil N, Cioffi G, Waite K, Kruchko C, Barnholtz-Sloan JS (2020) CBTRUS Statistical Report: Primary Brain and Other Central Nervous System Tumors Diagnosed in the United States in 2013-2017. Neuro Oncol 22: iv1-iv96. doi:10.1093/neuonc/noaa200

4. Louis DN, Perry A, Wesseling P, Brat DJ, Cree IA, Figarella-Branger D, Hawkins C, Ng HK, Pfister SM, Reifenberger G, Soffietti R, von Deimling A, Ellison DW (2021) The 2021 WHO Classification of Tumors of the Central Nervous System: a summary. Neuro Oncol 23: 1231-1251. doi:10.1093/neuonc/noab106

5. Goebel S, Stark AM, Kaup L, von Harscher M, Mehdorn HM (2011) Distress in patients with newly diagnosed brain tumours. Psychooncology 20: 623-630. doi:10.1002/pon.1958

6. Halkett GK, Lobb EA, Rogers MM, Shaw T, Long AP, Wheeler HR, Nowak AK (2015) Predictors of distress and poorer quality of life in HGG patients. Patient education and counseling 98: 525-532. doi:10.1016/j.pec.2015.01.002

7. Halkett GK, Lobb EA, Shaw T, Sinclair MM, Miller L, Hovey E, Nowak AK (2017) Distress and psychological morbidity do not reduce over time in carers of patients with high-grade glioma. Support Care Cancer 25: 887-893. doi:10.1007/s00520-016-3478-6

8. Halkett G, Lobb E, Shaw T, Sinclair M, Miller L, Hovey E, Nowak A (2018) Do carer’s levels of unmet needs change over time when caring for patients diagnosed with high-grade glioma and how are these needs correlated with distress? Support Care Cancer 26: 275-286.

9. Schaefer I, Heneka N, Luckett T, Agar MR, Chambers SK, Currow DC, Halkett G, Disalvo D, Amgarth-Duff I, Anderiesz C, Phillips JL (2021) Quality of online self-management resources for adults living with primary brain cancer, and their carers: a systematic environmental scan. BMC Palliat Care 20: 22. doi:10.1186/s12904-021-00715-4

10. Cancer Council Victoria, Department of Health Victoria (2021) Optimal care pathway for people with high-grade glioma. Melbourne

11. Centre for Improving Palliative; Aged and Chronic Care through Clinical Research and Translation (IMPACCT) (2020) Audit of National Care Standards, clinical pathways and decision support tools for patients with malignant brain cancer: Cancer Australia RFQ1819-02. Cancer Australia, New South Wales

12. Weller M, van den Bent M, Tonn JC, Stupp R, Preusser M, Cohen-Jonathan-Moyal E, Henriksson R, Rhun EL, Balana C, Chinot O, Bendszus M, Reijneveld JC, Dhermain F, French P, Marosi C, Watts C, Oberg I, Pilkington G, Baumert BG, Taphoorn MJB, Hegi M, Westphal M, Reifenberger G, Soffietti R, Wick W, European Association for Neuro-Oncology Task Force on G (2017) European Association for Neuro-Oncology (EANO) guideline on the diagnosis and treatment of adult astrocytic and oligodendroglial gliomas. Lancet Oncol 18: e315-e329. doi:10.1016/S1470-2045(17)30194-8

13. Eysenbach G (2004) Improving the quality of Web surveys: the Checklist for Reporting Results of Internet E-Surveys (CHERRIES). J Med Internet Res 6: e34. doi:10.2196/jmir.6.3.e34

14. Cooperative Trials Group for Neuro-Oncology (2018) Membership update. Member Newsletter. COGNO, Camperdown, NSW

15. Cancer Council Victoria (2015) Optimal cancer care pathway for people with high-grade glioma. Cancer Council Victoria, Cancer Council Victoria website

16. Fisher RA (1922) On the Interpretation of χ2 from Contingency Tables, and the Calculation of P. Journal of the Royal Statistical Society 85: 87-94. doi:<https://doi.org/10.1111/j.2397-2335.1922.tb00768.x>

17. Siegel C, Armstrong TS (2018) Nursing Guide to Management of Major Symptoms in Patients with Malignant Glioma. Semin Oncol Nurs 34: 513-527. doi:10.1016/j.soncn.2018.10.014

18. Bruhn H, Blystad I, Milos P, Malmstrom A, Dahle C, Vrethem M, Henriksson R, Lind J (2021) Initial cognitive impairment predicts shorter survival of patients with glioblastoma. Acta Neurol Scand -: -. doi:10.1111/ane.13529

19. Koekkoek JA, Dirven L, Sizoo EM, Pasman HR, Heimans JJ, Postma TJ, Deliens L, Grant R, McNamara S, Stockhammer G, Medicus E, Taphoorn MJ, Reijneveld JC (2014) Symptoms and medication management in the end of life phase of high-grade glioma patients. J Neurooncol 120: 589-595. doi:10.1007/s11060-014-1591-2

20. Pugh JD, McCoy K, Needham M, Jiang L, Giles M, McKinnon E, Heine K Evaluation of an Australian neurological nurse-led model of postdischarge care. Health & Social Care in the Community n/a. doi:<https://doi.org/10.1111/hsc.13498>

21. Riley J (2017) The Neuro Network Programme. Advances In Clinical Neuroscience & Rehabilitation 16: 19.

22. Weller M, van den Bent M, Hopkins K, Tonn JC, Stupp R, Falini A, Cohen-Jonathan-Moyal E, Frappaz D, Henriksson R, Balana C, Chinot O, Ram Z, Reifenberger G, Soffietti R, Wick W, European Association for Neuro-Oncology Task Force on Malignant G (2014) EANO guideline for the diagnosis and treatment of anaplastic gliomas and glioblastoma. Lancet Oncol 15: e395-403. doi:10.1016/S1470-2045(14)70011-7

23. Cancer Council Australia (2020) High-grade glioma cancer optimal cancer care pathway. Cancer Council Australia,

24. Australian Cancer Network Adult Brain Tumour Guidelines Working Party (2009) Clinical Practice Guidelines for the Management of Adult Gliomas: Astrocytomas and Oligodendrogliomas. Cancer Council Australia, Australian Cancer Network and Clinical Oncological Society of Australia Inc., Sydney

25. Butterbrod E, Synhaeve N, Rutten GJ, Schwabe I, Gehring K, Sitskoorn M (2020) Cognitive impairment three months after surgery is an independent predictor of survival time in glioblastoma patients. J Neurooncol 149: 103-111. doi:10.1007/s11060-020-03577-7

26. Coomans MB, van der Linden SD, Gehring K, Taphoorn MJB (2019) Treatment of cognitive deficits in brain tumour patients: current status and future directions. Curr Opin Oncol 31: 540-547. doi:10.1097/CCO.0000000000000581

27. Ownsworth T, Chan RJ, Jones S, Robertson J, Pinkham MB (2021) Use of telehealth platforms for delivering supportive care to adults with primary brain tumors and their family caregivers: A systematic review. Psychooncology 30: 16-26. doi:10.1002/pon.5549

28. Kevin Suen K-F, Tat Ming Chan D, Ho-Fung Loong H, Cw Wong K, Wm Yeung E, Cm Lam D, Chi-Ping Ng S, Yp Hsieh S, Ky Lau C, Yf Chan Y, Sm Fook S, Sang Poon W (2021) Health-related quality of life of glioblastoma patients receiving post-operative concomitant chemoradiotherapy plus adjuvant chemotherapy: A longitudinal study. Interdisciplinary Neurosurgery 26: 101339. doi:10.1016/j.inat.2021.101339

29. Rubenstein MF, Schenke-Reilly B (2018) Communication and swallowing impairments in brain cancer. Central Nervous System Cancer Rehabilitation: 63-73. doi:10.1016/B978-0-323-54829-8.00007-X

30. Kim WJ, Novotna K, Amatya B, Khan F (2019) Clinical practice guidelines for the management of brain tumours: A rehabilitation perspective. J Rehabil Med 51: 89-96. doi:10.2340/16501977-2509

31. Yu J, Jung Y, Park J, Kim JM, Suh M, Cho KG, Kim M (2019) Intensive Rehabilitation Therapy Following Brain Tumor Surgery: A Pilot Study of Effectiveness and Long-Term Satisfaction. Ann Rehabil Med 43: 129-141. doi:10.5535/arm.2019.43.2.129

32. Faculty of the Royal Australasian College of Physicians (2021) Australasian Faculty of Rehabilitation Medicine. RACP. <https://www.racp.edu.au/about/college-structure/australasian-faculty-of-rehabilitation-medicine>. Accessed 19 October 2021

33. Halkett GKB, Cormie P, McGough S, Zopf EM, Galvão DA, Newton RU, Nowak AK (2021) Patients and carers' perspectives of participating in a pilot tailored exercise program during chemoradiotherapy for high grade glioma: A qualitative study. Eur J Cancer Care (Engl) 30: e13453. doi:10.1111/ecc.13453

34. Pace A, Dirven L, Koekkoek JAF, Golla H, Fleming J, Ruda R, Marosi C, Le Rhun E, Grant R, Oliver K, Oberg I, Bulbeck HJ, Rooney AG, Henriksson R, Pasman HRW, Oberndorfer S, Weller M, Taphoorn MJB, European Association of Neuro-Oncology palliative care taskforce (2017) European Association for Neuro-Oncology (EANO) guidelines for palliative care in adults with glioma. The Lancet Oncology 18: e330-e340. doi:10.1016/S1470-2045(17)30345-5

35. Wu A, Ruiz Colon G, Aslakson R, Pollom E, Patel CB (2021) Palliative Care Service Utilization and Advance Care Planning for Adult Glioblastoma Patients: A Systematic Review. Cancers (Basel) 13. doi:10.3390/cancers13122867

36. Fritz L, Dirven L, Reijneveld JC, Koekkoek JA, Stiggelbout AM, Pasman HR, Taphoorn MJ (2016) Advance Care Planning in Glioblastoma Patients. Cancers (Basel) 8. doi:10.3390/cancers8110102

37. Palliative Care Australia (2018) National Palliative Care Standards. Palliative Care Australia, Canberra, Australia

38. Fox J, Thamm C, Mitchell G, Emery J, Rhee J, Hart NH, Yates P, Jefford M, Koczwara B, Halcomb E, Steinhardt R, O'Reilly R, Chan RJ (2021) Cancer survivorship care and general practice: A qualitative study of roles of general practice team members in Australia. Health Soc Care Community n/a. doi:10.1111/hsc.13549

39. Aubin M, Vézina L, Verreault R, Simard S, Hudon É, Desbiens JF, Fillion L, Dumont S, Tourigny A, Daneault S (2021) Continuity of Cancer Care and Collaboration Between Family Physicians and Oncologists: Results of a Randomized Clinical Trial. Ann Fam Med 19: 117-125. doi:10.1370/afm.2643

40. Thamm C, Fox J, Hart NH, Rhee J, Koczwara B, Emery J, Milley K, Nund RL, Chan RJ (2021) Exploring the role of general practitioners in addressing financial toxicity in cancer patients. Support Care Cancer: 1-8. doi:10.1007/s00520-021-06420-5

41. Halkett GKB, Berg MN, Breen LJ, Cutt D, Davis M, Ebert MA, Hegney D, House M, Kearvell R, Lester L, Maresse S, McKay J (2018) Sustainability of the Australian radiation oncology workforce: A survey of radiation therapists and radiation oncology medical physicists. Eur J Cancer Care (Engl) 27: e12804. doi:10.1111/ecc.12804

42. Australian Bureau of Statistics (2021) Population. Australian Bureau of Statistics,. <https://www.abs.gov.au/statistics/people/population>. Accessed 11/03/2022 2022

43. Cancer Institute NSW (2018) Cancer Statistics NSW. Cancer Institute NSW. <https://www.cancer.nsw.gov.au/research-and-data/cancer-data-and-statistics/cancer-statistics-nsw#//analysis/incidence/>. Accessed 11 March 2022 2022

### **Statements & Declarations**

**Funding**

Davina Daudu was awarded a Cancer Council of WA Student Vacation Scholarship to contribute to data collection and analysis.

Georgia Halkett is a recipient of a Cancer Council of WA Research Fellowship.

This study was part of a larger project funded by a PdCCRS Cancer Australia grant.

Completion of this manuscript was supported by the BRAINS project funded by MRFF.

**Competing Interests**

*The authors have no relevant financial or non-financial interests to disclose.*

**Author Contributions**

Georgia Halkett, Elizabeth Lobb, Jane Phillips, Danette Langbecker, Meera Agar, Elizabeth Hovey, Rachael Moorin, and Anna Nowak contributed to the study conception and design. Material preparation, data collection and analysis were performed by Georgia Halkett, Melissa Berg and Davina Daudu. Critical feedback on data analysis and interpretation was provided by all authors. The first draft of the manuscript was written by Georgia Halkett and Melissa Berg and all authors commented on previous versions of the manuscript. All authors read and approved the final manuscript.

**Data Availability**

The datasets generated during and/or analysed during the current study are available from the corresponding author on reasonable request.

**Ethics approval**

This study was performed in line with the principles of the Declaration of Helsinki. Approval was granted by the Ethics Committee of Curtin University (HRE2018-0706).

**Consent to participate**

Informed consent was obtained online from all individual participants included in the study.
