# Supplementary material for: Supportive care of patients diagnosed with high grade glioma and their carers in Australia
Source: J Neurooncol. 2022 Apr 9;157(3):475–85. doi: 10.1007/s11060-022-03991-z (PMC8994178; doi:10.1007/s11060-022-03991-z)
Supplement: Supplementary file 3 — Supplementary file3 (DOCX 13 kb) [file 11060_2022_3991_MOESM3_ESM.docx]

# Supplements

## Supplement 1 Summary of the survey modifications in the pilot phase

The questionnaire was piloted in a subset the participant sample and participant feedback, patterns of drop out and missing responses, informed further modifications. In response to feedback, thirty-two questions in the pilot survey about the proportion of patients referred to various supportive services at specific timepoints during treatment were replaced by five simplified questions in the final survey which did not capture information about the timing of referrals. In a further two questions the answer options were modified. Demographic questions were not changed.

As a result of the changes, pilot participants did not complete four new questions about the proportion of patients referred to available supportive care services. Pilot participants did not complete one new question about the proportion of carers referred to available supportive services.
