# Supplementary material for: Supportive care of patients diagnosed with high grade glioma and their carers in Australia
Source: J Neurooncol. 2022 Apr 9;157(3):475–85. doi: 10.1007/s11060-022-03991-z (PMC8994178; doi:10.1007/s11060-022-03991-z)
Supplement: Supplementary file 4 — Supplementary file4 (DOCX 32 kb) [file 11060_2022_3991_MOESM4_ESM.docx]

# Supplements

## Supplement 3: Figure 1

Figure 1: At sites where each was service available, the proportion of HGG patients referred to spiritual and practical support services, complementary service providers, and support groups. Five participants completed the pilot survey without these items.

Supplement 3: Table 1: The proportion of patients diagnosed with HGG at HPs' sites who are given each of source of information

| What proportion of patients receiving treatment for HGG at your site are given the following types of information: | All are routinely given this information  n (%) | Most are routinely given this information  n (%) | About half are given this information  n (%) | Some are routinely given this information  n (%) | Very few are given this information  n (%) | Don’t know  n (%) | Total  N = 36 (100%) |
| --- | --- | --- | --- | --- | --- | --- | --- |
| Written information developed at state or national level (e.g. Cancer Council, eVIQ information) | 18 (50) | 7 (19) | 5 (14) | 3 (8) | 1 (3) | 2 (6) | 36 (100) |
| Information about online resources not developed by your site | 8 (23) | 3 (9) | 7 (20) | 7 (20) | 7 (20) | 3 (9) | 35 (100) |
| Written information specifically developed by your site | 10 (28) | 7 (19) | 1 (3) | 3 (8) | 13 (36) | 2 (6) | 36 (100) |
| Group information sessions run by your site | 4 (11) | 4 (11) | 3 (8) | 4 (11) | 16 (44) | 5 (14) | 36 (100) |
| Online information specifically developed by your site | 3 (9) | 2 (6) | 1 (3) | 4 (11) | 19 (54) | 6 (17) | 35 (100) |
| Written information developed by a pharmaceutical company or device manufacturer | 2 (6) | 2 (6) | 2 (6) | 5 (14) | 17 (49) | 7 (20) | 35 (100) |

## Supplement 4:

Table 1: The proportion of HPs who advise patients diagnosed with HGG to seek care from their general practitioner for common health issues

|  | N=36 (100%)^a^ |
| --- | --- |
| Pre-existing chronic conditions or comorbidities | 34 (94) |
| Social issues (e.g. National Disability Services Parking permit, insurance, driving) | 13 (36) |
| Psychological support or psychiatric issues | 7 (19) |
| Complications of their cancer (e.g. deep vein thrombosis) | 4 (11) |
| Symptoms of their cancer (e.g. headache, seizure management) | 4 (11) |
| Side effects of their cancer treatment | 1 (3) |
| Other^b^ | 2 (6) |
| Not applicable | 2 (6) |

^a^ multiple responses allowed

^b^ palliative care or referral if patients lived far from the hospital (n=1) or referral to community services (n=1)

## Supplement 5: Carer tables and figures

Table 1: Availability of specialist nursing, psychological, and social supportive care services for carers of patients diagnosed with HGG

| At your site can you refer a carer of a HGG patient for support from a… | Service is available  n (%) | On site provider  n (%) | External or private provider n (%) | This service is not available at my site  n (%) | Unsure, I do not refer to this service  n (%) | Total  N=35 (%) |
| --- | --- | --- | --- | --- | --- | --- |
| Social worker or welfare officer | 29 (83) | 29 (83) | 0 (0) | 3 (9) | 3 (9) | 35 (100) |
| Cancer care coordinator/ nurse navigator | 25 (71) | 25 (71) | 0 (0) | 8 (23) | 2 (6) | 35 (100) |
| General psychologist^a^ | 21 (62) | 13 (38) | 8 (24) | 9 (27) | 4 (12) | 34 (100) |
| Support group | 16 (46) | 10 (29) | 6 (17) | 15 (43) | 4 (11) | 35 (100) |
| Nurse practitioner^a^ | 7 (21) | 5 (15) | 2 (6) | 20 (59) | 7 (21) | 34 (100) |
| Other^a^ | 3 (14) | 2 (10) | 1 (5) | 3 (14) | 15 (71) | 21 (100) |

^a^ contains missing data

Figure 1: At sites where each is service available, proportion of carers of HGG patients referred to specialist nursing, psychological and social supportive care services. One participant completed the pilot survey without these items.

Table 2: Types of support groups available to carers of HGG patients at sites which can refer carers to support groups

|  | N=15 (100%) |
| --- | --- |
| Group for brain cancer patients and carers | 9 (60) |
| Group for HGG patients and carers | 4 (27) |
| Group for cancer patients (any diagnosis) and carers | 4 (27) |
| Group for carers of cancer patients (any diagnosis) | 3 (20) |
| Group for carers of HGG patients | 1 (7) |
| Group for carers of brain cancer patients | 1 (7) |

^a^ multiple responses allowed

Table 3: Proportion of carers of HGG patients advised to present to their general practitioner at various timepoints during HGG treatment

| At each of the following time points, what proportion of carers of HGG patients are advised to present to their general practitioner? | All or most are advised to visit their GP  n (%) | About half are advised to visit their GP  n (%) | Some or very few are advised to visit their GP  n (%) | Never advised to present to GP as carer is not receiving medical advice  n (%) | Don't know  n (%) | Total  n (%) |
| --- | --- | --- | --- | --- | --- | --- |
| Diagnosis of their loved one | 2 (6) | 4 (13) | 16 (50) | 4 (13) | 6 (19) | 32 (100) |
| During inpatient hospitalisation/s of their loved one | 2 (6) | 3 (9) | 17 (53) | 5 (16) | 5 (16) | 32 (100) |
| During outpatient care of their loved one | 4 (13) | 3 (10) | 16 (52) | 3 (10) | 5 (16) | 31 (100) |
| As needed at any point during care of their loved one | 8 (25) | 4 (13) | 12 (38) | 4 (13) | 4 (13) | 32 (100) |
